# Structure, Morphology, and Faceting of TiO_2_ Photocatalysts by the Debye Scattering Equation Method. The P25 and P90 Cases of Study

**DOI:** 10.3390/nano10040743

**Published:** 2020-04-13

**Authors:** Federica Bertolotti, Anna Vivani, Daniele Moscheni, Fabio Ferri, Antonio Cervellino, Norberto Masciocchi, Antonietta Guagliardi

**Affiliations:** 1 Dipartimento di Scienza e Alta Tecnologia, To.Sca.Lab & INSTM, Università dell’Insubria, via Valleggio 11, 22100 Como, Italy; avivani@uninsubria.it (A.V.); d.moscheni@uninsubria.it (D.M.); fabio.ferri@uninsubria.it (F.F.); 2 SLS, Laboratory for Synchrotron Radiation-Condensed Matter, Paul Scherrer Institut, 5232 Villigen, Switzerland; antonio.cervellino@psi.ch; 3 Istituto di Cristallografia & To.Sca.Lab., Consiglio Nazionale delle Ricerche, via Valleggio 11, 22100 Como, Italy

**Keywords:** titania, photocatalyst, nanocrystal morphology, Debye scattering equation, synchrotron X-ray diffraction

## Abstract

Characterization of functional nanocrystalline materials in terms of quantitative determination of size, size dispersion, type, and extension of exposed facets still remains a challenging task. This is particularly the case of anisotropically shaped nanocrystals (NCs) like the TiO_2_ photocatalysts. Here, commercially available P25 and P90 titania nanopowders have been characterized by wide-angle X-ray total scattering techniques. Synchrotron data were modelled by the reciprocal space-based Debye scattering equation (DSE) method using atomistic models of NC populations (simultaneously carrying atomic and nanoscale structural features) for both anatase and rutile phases. Statistically robust descriptors are provided of size, morphology, and {101} vs. {001} facet area of truncated tetragonal bipyramids for anatase, jointly to polymorph quantification. The effects of using the proper NC shape on the X-ray diffraction pattern are analyzed in depth through DSE simulations by considering variable bipyramid aspect ratios (resulting in different {101} vs. {001} surface) and relative dispersion in a bivariate manner. We demonstrate that using prismatic NCs having equal volume and aspect ratio as bipyramids provides reasonably accurate sizes and {101} and {001} surface areas of the parent morphology.

## 1. Introduction

The functionality of crystalline nanomaterials, and particularly of those possessing anisotropic shapes (as a consequence of low(er) symmetry structures or synthesis conditions), is heavily dependent on the surface extension and on the type of exposed crystal faces [1]. This widely encompasses, to mention a few, chemical reactivity, catalysis, rheology, luminescence, and photocatalytic activity [2,3].

**Figure 1:**
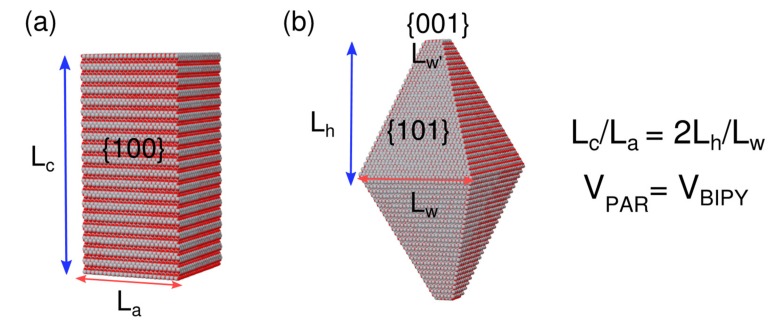
(**a**) A tetragonal square prism, characterized by *L_a_* and *L_c_* descriptors; (**b**) a tetragonal truncated square bipyramid, characterized by *L_w_*_,_ *L_w’_*_,_ and *L_h_* descriptors. Lateral faces in (**a**) are {100}, inclined faces in (**b**) are {101}, and basal faces are {001} in both polyhedra.

Titanium dioxide (TiO_2_), the “golden standard” among photocatalysts, exhibits properties that are strongly dependent on the structure, surface, and morphology of nanocrystals (NCs), because the photocatalytic reactions take place at the catalyst–substrate interface. With reference to anatase, the most photocatalytically active titania polymorph, it exhibits a tetragonal crystal structure [1], and surface energy calculations predict that a slightly truncated square bipyramid, exposing {101} and {001} facets only (see Figure 1), is the equilibrium shape (in the bulk), with {101} accounting for ~94% of the entire surface [2].

At the nanoscale, anatase is also reported to be the most stable polymorph below 30 nm [1]. Nevertheless, quantitatively defining the morphology and facet area and clarifying their role at this size regime, still remains a matter of deep exploration. NCs with extended {001} facets have been reported to be highly desirable, as the larger number of undercoordinated Ti atoms (active sites) present therein determine a higher reactivity if compared to the most stable {101} or {100} facets [4,5]. In order to induce the desired morphology with the stabilization of the high energy {001} facets, many attempts have been made by developing suitable synthetic routes, also considering the use of capping agents [6,7,8] or other surface stabilizers such as fluorine under hydrothermal conditions [5,9]. However, conflicting results have been reported in this regard, likely related to the difficulty of separately quantifying the activity of the different facets and the concurrence of surface hydroxylation (ubiquitous in low temperature syntheses), making the correlation between facets extent and photocatalytic properties still a debated issue [10,11,12].

Determining the correct size and shape distribution, that is the correct morphology of the entire nanoparticle ensemble, and providing the robust statistical estimation of model parameters, is difficult (for TiO_2_ and other nanosystems as well) by imaging techniques, like transmission electron microscopy (TEM), either in conventional or high-resolution (HR-TEM) mode. Indeed, the statistical robustness of such measurements is limited by the paucity of NCs which can be observed (in a reasonable amount of time) as well as by the fact that 2D projections are normally imaged, with intrinsic loss of the crystal thickness down the observation axis [5].

In recent years, this challenging task has been tackled by the use of advanced scattering/diffraction techniques (mostly using hard X-rays) where the beam, impinging on macrosized (mm^3^) powdered volume samples (like in conventional powder diffraction) enables wide-angle X-ray data to be collected in total scattering mode (WAXTS). With these techniques, both Bragg and diffuse scattering (originating from finite-size and structural defects) are measured and data are amenable to both real space (in pair distribution function, PDF, mode) [13,14] and reciprocal space total scattering analysis (through the Debye scattering equation, DSE) [15,16].

Advanced PDF and DSE methods provide robust tools for the physicochemical characterization of engineered nanomaterials. Though similar, the two approaches have progressed over recent years (at dedicated synchrotron facilities) in a parallel though different manner, by optimizing their own experimental setups and computational tools, which make one method highly complementary to the other. The DSE method, enabling calculation of the WAXTS trace of any kind of nanomaterials, was used by us, and others, to derive statistically sound ensemble properties. These include size and shape average values and variances, aspect ratio, faceting, and structural defectiveness, for a large variety of either pure crystalline nanophases or complex hybrids of technologically relevant classes of materials (semiconductors, oxides, perovskites, bioceramics, and metals), including TiO_2_ nanoparticles prepared by sol–gel synthesis [17,18,19,20,21,22,23,24,25,26].

With reference to X-ray total scattering analysis of nanosized TiO_2_, worth to mention is a recent study by Page and coworkers [5], reporting an in-depth characterization of pure nanoanatase with controlled size, morphology, and {101} vs. {001} surface ratios, based on the combination of differential evolution algorithms and DSE and PDF analyses. Using the proper bipyramidal morphology (predetermined through TEM imaging) for anatase NCs, WAXTS (real and reciprocal space) data analysis allowed important advances in the understanding of structure–photocatalytic property correlations to be achieved. Interestingly, the study demonstrated that the coexistence of {101} with {001} facets in intermediate proportions results in a higher photocatalytic activity for the hydrogen evolution reaction, if compared to samples with largely predominant {001} or {101} facets [5].

Commercial pyrogenic titania samples, marketed under the P25 and P90 labels, have been widely investigated to understand the relationship between structure, size, and their excellent photocatalytic properties [27,28,29,30,31,32,33,34,35,36]. The presence of a small amount of rutile nanoparticles (10–15% by weight) in these materials has been suggested to have an active role, serving as “antenna” or photosensitizer extending the anatase band gap and thus improving its photocatalytic activity [37,38,39]. Specific examination of the relevant bipyramid facets of anatase NCs in P25 and P90 has been recently performed through pair distribution function analysis using monodisperse morphological models [39]. Results have demonstrated that a crystal shape having ~20% of {001} surface is the best candidate for anatase. The study further reported on the size similarity (contradicting previous reports) between the majority (anatase) and minority (rutile) phases.

Taking inspiration from these recent works, in the present paper, we investigate the feasibility of retrieving accurate morphological and faceting details of truncated square bipyramidal anatase NCs from DSE analysis of WAXTS data, considering size and shape polydispersity of NCs within a bivariate modeling approach. We demonstrate that the main morphological information of (even polydisperse) NCs can be retrieved using either bipyramidal or prismatic models of identical volume and aspect ratio and that bipyramid size and faceting can be extracted from the prismatic approximation with reasonable accuracy. We then discuss the analysis of WAXTS data collected on the P25 and P90 powders at the Materials Science Beamline of the Swiss Light Source [40], where a forefront protocol for DSE-based analysis of engineered nanomaterials has been developed. The DSE modeling was performed using bivariate populations of (001) truncated square bipyramids for anatase (P90) or, alternatively, a bivariate population of tetragonal prismatic NCs, when the large NC sizes and 2D polydispersity made the analysis computationally too demanding (P25). Finally, results from the DSE-based approach are compared to those obtained using conventional Rietveld analysis.

## 2. Materials and Methods

### 2.1. Synchrotron Wide-Angle X-Ray Scattering (WAXTS) Data Collection and Reduction

Nanosized powders of the P25 (Sigma-Aldrich, St. Louis, MO, USA) and P90 (Evonik Aeroxide TiO_2_ P90, Hanau-Wolfgang Germany) titania composites were obtained from commercial sources and used as received. Synchrotron X-ray diffraction measurements were performed on sealed capillaries (Φ = 0.5 mm) of glass type 0500 from Hilgenberg GmbH, filled with the P25 or P90 powder at the MS-X04SA beamline of the Swiss Light Source (Paul Scherrer Institut, Villigen, Switzerland) [40]. The operational wavelength for a nominal 17.5 keV X-ray beam was set at λ = 0.709292 Å and calibrated through the use of Si SRM 640d (Nation Institute of Standards and Technology (NIST) Standard), with certified a_0_ = 5.43123(8) Å cell parameter. Wide-angle X-ray scattering (WAXTS) data were collected in transmission mode in the 2–120° 2*θ* range using a single-photon counting silicon microstrip MYTHEN II detector [41]. Separate air and empty capillary scattering measurements were performed, and the linear absorption coefficients of the samples were estimated through “radiographic” measurements (attenuated direct beam with and without the sample), while the one for the empty capillary was calculated from the glass certified composition and the tables provided by NIST website. The transmission of each specimen was numerically estimated using an X-ray tracing approach [42,43]. Raw data were then corrected for systematic errors (zero position and offsets) and absorption effects; the extra-sample contributions to the diffraction pattern, namely, the empty capillary and the sample environment scattering, were properly subtracted. Eventually, the final corrected data only account for the sample contribution to the diffraction pattern, which was analyzed through the total scattering approach based on the Debye scattering equation.

### 2.2. The Debye Scattering Equation Approach

The total scattering method here applied relies on the use of the Debye scattering equation (DSE) [44], enabling the computation of diffraction patterns of randomly oriented nanoparticles from the distribution of interatomic distances within the sample, without any assumption on the structural order. The simulated pattern is calculated according to the Formula (1):

(1) $$I\left(Q\right)=\overset{N}{\underset{j=1}{\sum }}f_{j}{\left(Q\right)}^{2}o_{j}{}^{2}+2\overset{N}{\underset{j>i=1}{\sum }}f_{j}\left(Q\right)f_{i}\left(Q\right)T_{j}\left(Q\right)T_{i}\left(Q\right)o_{j}o_{i}\frac{sin\;\left(Qd_{ij}\right)}{\left(Qd_{ij}\right)}$$

where *Q = 4*πsin*θ/λ* is the scattering vector amplitude, *θ* is half of the scattering angle 2*θ, λ* is the radiation wavelength, *f_j_* is the atomic form factor of atom *j*, *d_ij_* is the interatomic distance between *i* and *j* atom pairs, and *N* is the number of atoms in the nanoparticle. *T* and *o* refer to the isotropic atomic thermal vibration and site-occupancy parameters, respectively. The first summation accounts for the contribution of the zero distances of each atom from itself, whereas the second summation accounts for the non–zero distances between pairs of distinct atoms. The DSE analysis of WAXTS data collected on P90 and P25 was herein carried out using the DebUsSy Suite through subsequent steps, following a bottom-up approach [45]. At first, for each sample, the anatase and rutile cell parameters (*a* = *b* and *c*) were determined through the Rietveld refinement method implemented in the TOPAS Academic software [46], using a spherical harmonics model to phenomenologically describe the finite-size broadening of diffraction peaks (vide infra).

The derived unit cells were then used as building blocks to generate populations of atomistic models of NCs of increasing size. For anatase (the majority phase of P90 and P25), two distinct morphologies were considered (to be used in separate tests), both matching a bivariate population of NCs: *i*) a square prismatic shape with NCs grown along two perpendicular (independent) directions, one along the *c* axis and one in the *ab* plane [18,45] and *ii*) a truncated tetragonal bipyramid, constructed according to the algebraic expressions presented in Section 2.3. For rutile (the minority phase), a univariate population of NCs of spherical shape and growing diameter D was used.

Following the typical Debussy run, for each population, sampled interatomic distances of all pertinent NCs were encoded in a database and subsequently used to feed the DSE for calculating the diffraction pattern. The “sampled distances” were obtained through a sampling algorithm of the true distances able to reduce by order of magnitude the number of terms in the DSE without losing accuracy in the calculated pattern [47]. This trick made the analysis feasible in terms of computational time. The calculated diffraction pattern was then compared to the experimental one and the corresponding χ^2^ function was minimized (through the Simplex algorithm) by refining a number of adjustable structural and microstructural parameters in the model [48]. Size and anisotropic shape of the anatase NCs were described according to five parameters of a discrete bivariate lognormal distribution function. Taking the *c* axis and the *ab* plane as the two independent orthogonal NCs growth directions, the five parameters of the lognormal distribution consist of two pairs of average size and standard deviation, one for each growth direction, (*D_ab_*, *σ_ab_*) and (*L_c_*, *σ_c_*), and the inclination angle *φ* between them (indirectly measuring the correlation between the two growth axes). The standard deviations *σ_ab_* and *σ_c_* describe the size dispersions along the growth direction [18]. Nanomaterials exhibiting relative dispersions (*σ_ab_/D_ab_*, *σ_c_/L_c_*) up to 20% are typically reported as monodisperse; larger values indicate polydisperse sizes. Details of the model parameters optimization and refinement procedure for P90 and P25 DSE-based analysis are provided in the pertinent section.

Code availability: The DebUsSy program suite is freely available at http://debussy.sourceforge.net.

### 2.3. Geometrical Mapping of Square Prisms and Truncated Square Bipyramids of D_4h_ Symmetry

The common way atomistic descriptions of nanocrystal structure and stoichiometry are built, uses sequential growth in one (radial, *r*) or more (*x, y, z*) dimensions of starting building blocks of simple shape, typically related to the crystal unit cell, or, occasionally (for centered cells), their primitive analogues of lower symmetry. In the present study, for the tetragonal cells of anatase, NCs were grown along two independent directions (along *a* and *c* unit cell axes, respectively), describing *n_a_^2^* × *n_c_* cells in progressively larger square prisms (rectangular parallelepipeds), characterized by *L_a_* = *n_a_* × *a* basal edge, *L_c_* = *n_c_* × *c* height, and an aspect ratio *AR_P_* = *L_c_/L_a_*. These prisms, each of volume *V_P_* = *L_a_^2^ × L_c_*, can be built for any *n_a_*, *n_c_* combination, provided that *n_a_*, *n_c_* are positive integers. Figure 1a pictorially shows the pertinent geometrical entities.

As discussed in the introduction, anatase is known to occur as truncated square bipyramids with {101} lateral faces (inclined ca. 68.3° = tan^−1^ (*c/a*) from the equator) substituting {100} faces of the prism. The morphological descriptors are then *L_w_* (the edge of the equator), *L_w’_* (the edge of the basal {001}), and *L_h_* (the height of the (one sided) pyramid). With these definitions in mind (depicted in Figure 1b), the bipyramid volume is *V_B_* = 2/3*L_h_(L_w_^2^ + L_w’_^2^ + L_w_L_w’_)* and its aspect ratio is *AR_B_* = *2L_h_/L_w_*.

Mapping of prisms to bipyramids can be done imposing equivalence of aspect ratios (*AR_P_* = *AR_B_*) and polyhedral volumes (*V_PAR_* = *V_BIPY_*), using the algebraic relations shown in Equations (2) and (3), where *k* = *2a/c*.

(2) Lw= 123 La5312La2−6kLaLc+k2Lc23

(3) Lh= 323 La23Lc12La2−6kLaLc+k2Lc23

Accordingly, any bivariate population of tetragonal prisms (*L_a_, L_c_*) can be mapped into a new population of tetragonal truncated bipyramids (*L_w_, 2L_h_*), with the caveat that, ensuring convex polyhedra, a limitation of the 2*L_h_/L_w_* value must be set equal to *c/a* ≈ 2.5. This, in turn, means that prismatic NCs with large aspect ratios cannot be mapped into suitable convex bipyramids.

## 3. Results and Discussion

### 3.1. Influence of Nanocrystals (NCs) Morphology and Facets Surface Area on the WAXTS Pattern of Anatase

Among those NCs which are well known to adopt a clear morphology with non–orthogonal faceting, titania, in the form of anatase, is a paradigmatic case. As anticipated, slightly truncated square bipyramids, exposing {101} and {001} facets, are predicted to be the equilibrium shape in bulk anatase and are frequently imaged also at the nanoscale [7,49].

**Figure 2:**
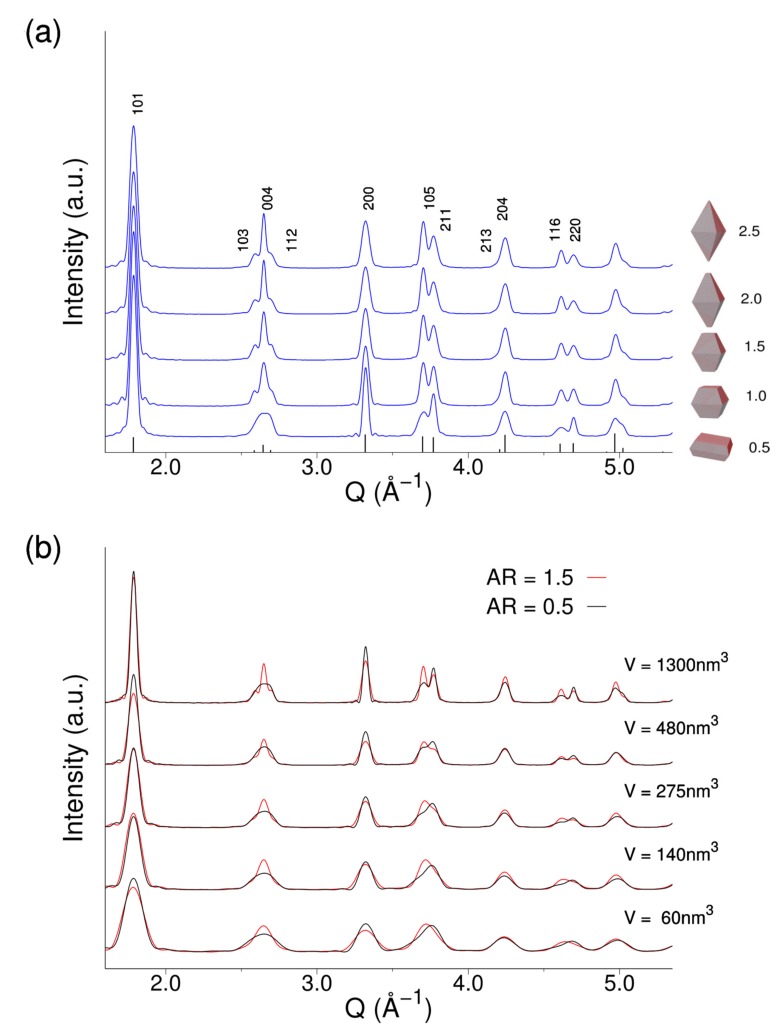
Simulated X-ray scattering patterns for anatase nanocrystals (NCs) having truncated bipyramidal shapes, (**a**) with different aspect ratios, *AR*, at constant volume (1300 nm^3^) and (**b**) with different volumes at constant *AR* = 0.5 and *AR* = 1.5. Simulations in (**a**) are on the absolute scale, whereas in (**b**) scaling was applied to facilitate the comparison of nanocrystals with different volumes.

In Figure 2a, we encode the most relevant effects of such morphology on the X-ray diffraction peaks upon varying the bipyramid aspect ratio (*AR = 2L_h_/L_w_*) at constant nanocrystal volume. Significantly, by “squeezing” the bipyramids from *AR* = 2.5 to *AR* = 0.5, which leads to the progressive widening of the {001} facets at the expenses of the {101} ones, the triplet at *Q* = 2.6 Å^−1^ and the doublets at *Q* = 3.7 Å^−1^ and *Q* = 4.7 Å^−1^ are markedly affected, being Bragg peaks in those regions associated to crystal directions aligned nearly along the *c* axis (103, 004, 112, 105, and 116 reflections) or in the *ab* plane (200, 220, and 211 reflections). DSE simulations in Figure 2b encode the Bragg peak changes at identical *AR*, but progressively increasing NC volumes from 60 to 480 nm^3^ (linear sizes falling approximately in the 2–5 nm range) in either markedly flat (*AR* = 0.5) or stretched (*AR* = 1.5) bipyramids.

However, a comparable dependence of peak widths and shapes is obtained if prismatic anatase NCs of equal volume and same *AR* are used in the DSE calculations. Therefore, it is highly conceivable that, in the presence of smearing effects due to size and shape polydispersity, those tiny faceting details introduced by bipyramids may not be properly captured nor accurately quantified. On the other side, whether and to which extent, approximating the predicted bipyramidal crystal shape of anatase NCs, by simple square prisms oversimplifies the extraction of relevant size and morphological parameters through WAXTS analysis, is unknown.

In order to clarify this issue, we simulated by DSE a number of WAXTS patterns from bivariate populations of atomistic models of anatase NCs having a real bipyramidal shape (labelled as BIPY hereafter) with controlled *<AR>* = *<2L_h_>/<L_w_>* and size and shape polydispersity (depending on *σ_h_/2L_h_*, *σ_w_/L_w_* and the correlation angle *φ_B_* between the two growth directions), to which a Poisson noise comparable to that of P90 and P25 synchrotron data (signal/noise ~700) was added. These synthetic “experimental” data were fitted following the standard DebUsSy approach, using a bivariate lognormal population of prisms through optimization of a number of model parameters (given in Table A1) against the synthetic data [18]. The 2D size distribution map from the best pattern match (according to the minimum GoF value; GoF = √χ^2^) was then converted from prims to bipyramids (labelled as BIPY_PAR_ hereafter) using the algebraic expressions described in Section 2.3.

**Figure 3:**
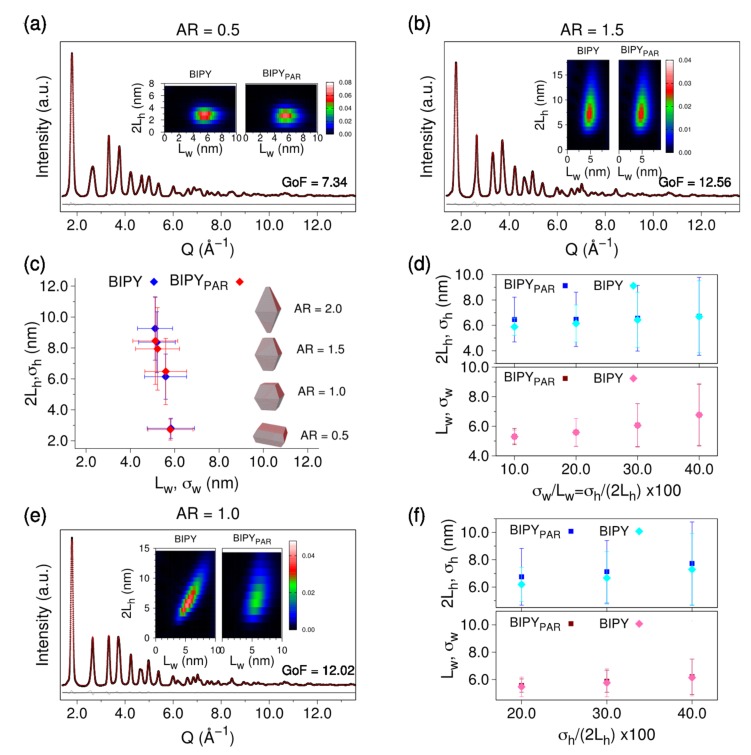
Analysis of X-ray pattern simulations from anatase bipyramids (BIPY) NCs fitted using prismatic NCs, from which relevant parameters of the parent prismatic morphology (BIPY_PAR_) are extracted. (**a**,**b**,**e**) BIPY simulations (black dots, Poisson noise with S/N ≈ 700), best DSE fits (red traces) obtained using a bivariate population of tetragonal prisms by adjusting size and shape parameters. As these curves closely superimpose, the absolute residual trace (in gray), shown at the bottom, addresses the tiny mismatch between them. BIPY simulations correspond to <*AR> = <2L_h_>/<L_w_>* = 0.5 (**a**), <*AR>* = 1.5 (**b**), <*AR>* = 1.0 (**e**), 20% relative dispersion (σ*_h_/<2L_h_ > =* σ*_w_/<L_w_> =* 0.2*, φ_B_* = 0°) along the two growth directions in (**a**,**b**), and σ*_h_/<2L_h_> =* 0.4*,* σ*_w_/<L_w_> = 0.1, φ_B_* = 30° in (**e**). Insets in (**a**,**b**,**e**) show the color-encoded (mass based) BIPY 2D maps and those recovered by the BIPY_PAR_ model; (**c**) comparison of average size, shape, and dispersion parameters of BIPY at increasing <*AR>* (blue diamonds) with BIPY_PAR_-retrieved values (red diamonds) and fixed polydispersity. Horizontal and vertical bars represent the standard deviations of the lognormal distributions (σ*_h_ =* 0.2*<2L_h_>* and σ*_w_ =* 0.2*<L_w_>* in the BIPY population). The numerical outcomes of this plot are synoptically reported in Table A1; (**d**,**f**) BIPY and BIPY_PAR_ size and size dispersion parameters at increasing σ*_h_/<2L_h_>* values, at *φ_B_* = 0° (**d**) and *φ_B_* = 30° (**f**). In both panels, results about *L_w_*, *σ_w_* from BIPY_PAR_ models nearly superimpose onto the BIPY ones.

Synthetic WAXTS patterns were simulated using BIPY NCs with different *<AR>* (0.5, 1.0, 1.5, and 2.0), at fixed polydispersity (*σ_h_/2L_h_* = *σ_w_/L_w_* = 20%) and *φ_B_* = 0° (meaning that 2 *L_h_* and *L_w_* are fully uncorrelated). Figure 3a,b shows the best fits at markedly distinctive *AR* (0.5 and 1.5). Color-coded 2D maps (insets) depict the mass-based size distributions of the “true” BIPY and converted BIPY_PAR_ populations. While GoF values (measuring the agreement of pattern matching) are well above one, nevertheless the relevant size and shape parameters of the BIPY_PAR_ model, and their distributions, are reasonably well reproduced, as shown in Figure 3c for the different *<AR>* (and numerically reported in Table A1). Worth of note, the prismatic model is better performing at lower *<AR>* than for more elongated NCs, as demonstrated by the lower GoF value (7.34 vs. 12.56) and by the closer similarity between the average sizes and size dispersions of the starting BIPY bipyramidal and recovered BIPY_PAR_ values, as shown in Figure 3c. Nevertheless, we found that *L_a_* and *L_w_* are nearly identical, suggesting that the *AR* has minor influence on the equatorial BIPY size, while in the axial direction, discrepancies between *L_c_* and 2 *L_h_* values increase at larger *AR,* reaching the maximum deviation (<1 nm) at *AR* = 2.0, for the selected relative size distribution of 20%.

Being aware of the well-known smearing effects of larger size distributions on WAXTS-based analysis, additional BIPY simulations were considered at fixed *<AR>* = 1.0 and larger polydispersity. Figure 3d shows the recovered BIPY_PAR_ values at *σ_h_/2L_h_* = *σ_w_/L_w_* = 10%, 20%, 30%, and 40% (*φ_B_* = 0°); Figure 3f displays the recovered BIPY_PAR_ values at *σ_w_/L_w_* = 10% ≠ *σ_h_/2L_h_* = 20%, 30%, and 40% (*φ_B_* = 30°). The best fit and 2D size distribution map insets in Figure 3e refer to the case with the largest dispersion in the axial direction (*σ_h_/2L_h_* = 40%) and correlation between the two growth axes (*φ_B_* = 30°). Results in Figure 3d,f (numerically quoted in Supplementary Tables S1 and S2) suggest that for nearly isotropic BIPY (*AR* close to 1): *i*) *L_w_* values and the relative polydispersity are fully recovered by the BIPY_PAR_ model, regardless of the size dispersion; *ii*) *2L_h_* shows very minor deviations (within 10% and decreasing upon raising the polydispersity); and *iii*) *σ_h_/2L_h_* are slightly overestimated to a maximum 15% level.

Worth noting, when the BIPY model is used to fit the synthetic data shown in Figure 3a,b,e, GoF values close to 1 are obtained as expected for an unbiased model (in the presence of statistical noise only), the accuracy of recovered parameters (tested for different starting values for each case) remaining within 0.1%.

### 3.2. Analysis of P25 and P90 WAXTS Data by Conventional Rietveld Analysis

Conventional Rietveld analysis was performed on both P25 and P90 datasets, taking the structural models from refs. [50] and [51], for anatase and rutile, respectively. With this approach, parallel refinements were performed using isotropic and anisotropic peak width models, the latter being treated by a fourth-order spherical harmonics model (for anatase only).

Results are summarized in Table 1 for the two models and compared with nominal values taken from material data sheets provided by the vendors. Treating the peak width anisotropy in Rietveld refinement improves the quality of the fit (lowering the values of all statistical indicators) without significantly affecting the size estimations. The reported sizes of anatase (ca. 15 nm in P90 and 26.0 nm in P25, not containing size dispersion information) are quite in line with the nominal ones (from TEM imaging, single size, 14 and 21 nm in P90 and P25, respectively). The small deviations may be reasonably attributed to intrinsic technique-driven differences, considering that number-based and volume-based averages are provided by TEM and XRD analyses, respectively. The minority rutile phase is quantified in comparable amounts (~10% by weight) in both P25 and P90. The rutile NC sizes are much smaller in P90 (23 nm) than in P25 (45 nm). Final Rietveld refinement plots are provided as Supplementary Figure S1 together with the average crystal shapes of anatase NCs drawn on the basis of the spherical harmonic coefficients.

### 3.3. Analysis of P25 and P90 WAXTS Data through the Debye Scattering Equation (DSE)

The smaller average size of NCs in P90 (compared to the P25 case), allowed the DSE-based analysis of this sample to be performed by using both the BIPY and the BIPY_PAR_ models for anatase, enabling the results from real experimental data to be compared for the two models. The final bivariate BIPY population contains all possible *2L_h_*, *L_w_* combinations (within the *AR* limit of 2.5 described in Section 2.3), up to sizes as largest as 30 nm in the two NC growth directions. The bivariate population of prisms was built independently, using the same size discretization (by adding layers of unit cells, using the same lattice parameters as in BIPY, see Table 1). In contrast, for P25, only the BIPY_PAR_ model was applied. In both P90 and P25 samples, the rutile minority phase was described by spherical NCs.

The model parameters optimized against the experimental data (Table 2) include structural parameters (the isotropic Debye–Waller factor, *B*, for all atoms and site occupancy factor, *s.o.f.,* for Ti), average size, and standard deviation along each of the two growth directions and their correlation angle, according to a bivariate lognormal distribution. For rutile, the average diameter and its standard deviation were also refined (assuming a univariate lognormal distribution). Finally, a tiny microstrain effect (ε = 0.2% in P90, ε = 0.1% in P25, based on a phenomenological model) was introduced to account for additional peak broadening at high angle (unrelated to finite-size effects) [52].

**Figure 4:**
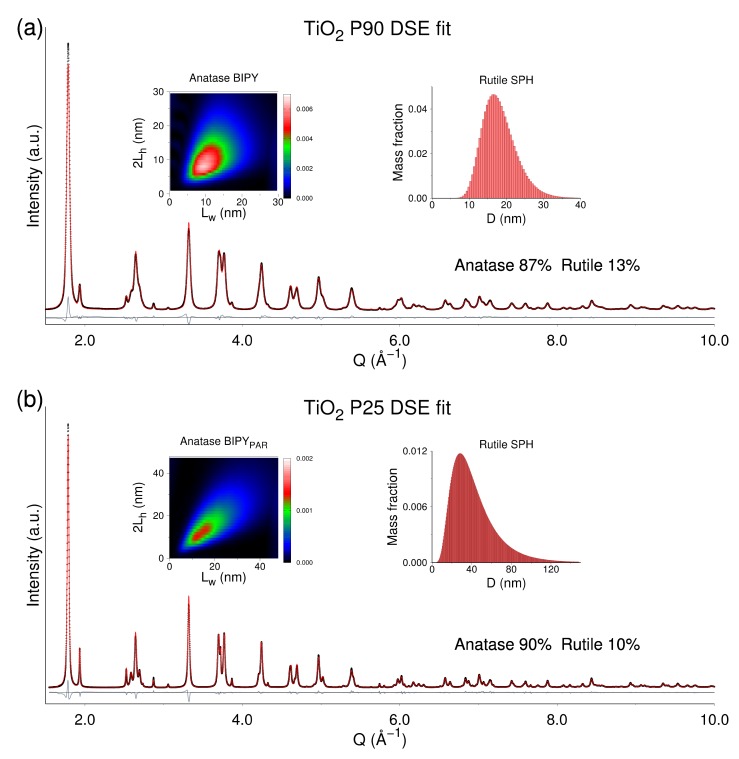
Wide-angle X-ray scattering (WAXTS) data (black dots) and best fits (red curves) provided by the DSE-based method using a bivariate population of (**a**) bipyramids (BIPY) for anatase in P90 and (**b**) square parallelepipeds (PAR) for anatase in P25, and a univariate population of spherical NCs for rutile in both samples. Insets in (**a**) and (**b**) (left): 2D maps of the bivariate lognormal (mass-based) size distribution (in the *L_w_*, 2*L_h_* coordinates) of anatase NCs (BIPY in P90 and BIPY_PAR_ in P25) and (right): (mass-based) univariate lognormal size distribution of rutile NCs.

Results of the BIPY (P90) and BIPY_PAR_ models (P90 and P25) are synoptically collected in Table 2. Figure 4 visualizes the final best matching between the experimental and DSE-calculated data for P90 and P25, respectively; the color-coded (mass-based) bivariate 2D size distribution of the anatase fraction and the histograms of the univariate lognormal distribution are used for rutile (insets).

Very minor differences are found among BIPY- and BIPY_PAR_-derived parameters for P90, confirming the findings of simulated data. Apparently, the major advantage of using true bipyramids results in a better pattern matching (lowering the GoF down to 11.57 from 12.12), suggesting that the tiny details of faceting are indeed encoded in the experimental data. Nevertheless, major morphological information, such as *AR*, sizes, and surface areas of {101} vs. {001} facets, are recovered by the prismatic approximation with good accuracy, likely due to polydispersity effects. On this basis, also considering the similarity between P25 and P90 results emerging from our analysis (if we neglect the larger NCs size of the former), in the following considerations, we will refer to the outcome from the BIPY_PAR_ model for both samples, without further specifications.

Bipyramids in P90 are smaller by about 60% in the axial direction and by 64% in the equatorial one than those in P25. This leads to nearly isotropic anatase NCs in both samples, though slightly more elongated in P90 (*AR* = 0.95) than in P25 (*AR* = 0.89), in line with Rietveld analysis (*AR* = 0.85 vs. *AR* = 0.80, Table 1). Moreover, considering the comparable high-size dispersion (approaching 50% along the two growth directions), the surface area of the more active {001} facets (averaged through the whole population of NCs) in P90 (20.8%) is slightly smaller than in P25 (22.7%), in agreement with previous studies [39]. Rutile is ca. 30% larger than anatase in P90 (~18 vs. 14 nm, see Table 2) and much larger (ca. 75%) in P25. The quantification of the rutile component, 10.4% by weight in P25, well matches that from Rietveld analysis (11.6 wt%), and both are well in agreement with previous reports [39]. In P90, a slightly larger amount (13.1 wt%) is found, larger than the Rietveld estimation (8.2 wt%). This mismatch may reasonably be attributed to differences in the modeling approach (the Rietveld method disregards diffuse scattering due to finite-size effects, with influences on the background level that are more effective in P90). Our modeling also confirms that anatase and rutile coexist as a biphasic mixture in P90 and P25 samples, and from the structural point of view, there is no reason to rely on more complex “intergrowth” structure of the two polymorphs [53].

With reference to the photocatalytic properties of the two titania samples, the well-documented higher performance of P90 well matches the smaller average sizes of the constituent nanoparticles, resulting in larger specific surface area (SSA, in an approximate 2:1 ratio) [54,55,56,57,58,59]. As reference, we provide in Table 2, the SSA calculated for the two samples. Though the absolute values are overestimated by the DSE modeling (which disregards particle aggregation), their ratio is in line with that derived from nitrogen gas adsorption measurements. Values claimed by the vendors are 90 ± 20 m^2^·g^−1^ (P90) and 50 ± 15 m^2^·g^−1^ (P25) [60]. However, it is reported that the activity of P90 normalized to the surface area is lower than that of P25 [61] (see Table S3 for the referenced values). On the basis of our DSE analysis, we tentatively attribute this fact to the slightly more “squeezed” morphology of anatase in P25, in which NCs exhibit less unbalanced, optimized proportions of {001} and {101} surfaces, in agreement with the results reported by Liu et al. [5].

Concerning the role of rutile on the different activity of the two photocatalysts, we note that the quantification from our DSE analysis conflicts with most of the literature reports that provide larger fractions in P25 than in P90 (in line with our Rietveld outcome) [39,61]. Many papers support the synergic rutile/anatase effects in the activity of the mixed-phase TiO_2_ photocatalysts [4,53,62,63]. Whether the rutile amount, and size (dis)similarity between the two polymorphs as well, may have a direct influence in such a synergy cannot be answered just on the basis of the results presented in this work. Combining the DSE-based analysis with dedicated experiments might further help in addressing this issue.

## 4. Conclusions

In summary, we have presented an in-depth structural and morphological characterization of commercial P25 and P90 nanosized photocatalysts. Using synchrotron wide-angle X-ray total scattering data, we applied a forefront analysis in reciprocal space, relying on atomistic models of anatase and rutile polymorphs and the Debye scattering equation. For anatase, a population of NCs having anisotropic shapes was considered within a bivariate model using either square bipyramids, exposing {101} and {001} facets, or prismatic shapes. We have demonstrated that the bivariate population of bipyramids can be mapped into prismatic NCs having equal volumes and aspect ratios. Using such an approximation, relevant morphological parameters of bipyramids can be recovered with good accuracy as robust statistical indicators of average size, polydispersity, aspect ratio, and {101} vs. {001} surface areas. The comparative analysis of P25 and P90 data suggests that {001} facets in the former are slightly more represented, which may explain the reported higher photocatalytic activity once normalized to surface area. The method here proposed for P90 and P25 can be extended to describe the bipyramidal morphology of anatase NCs in titania samples of different origin. In case of NCs having AR > 2.5 and morphologies more complex than bipyramids (due to the appearance of {100} facets), the prismatic approximation is considered even more appropriate.

## Figures and Tables

**Table 1:** Synoptic results of the conventional Rietveld refinement approach to size/shape determination, obtained by TOPAS according to an isotropic (*Iso*) and anisotropic (*Aniso*) morphological description of anatase. A single isotropic Debye–Waller factor was refined for all atoms in P25 (B = 0.53 or 0.49 Å^2^) and P90 (B = 0.58 or 0.51 Å^2^; for the *Iso* and *Aniso* models, respectively). *<CS>* = average crystal size; *M* and *N* subscripts = mass and number based.

Parameter	P90		P25	
	Anatase	Rutile	Anatase	Rutile
*a, c* (Å)	3.7863, 9.4985	4.5947, 2.9592	3.7860, 9.5080	4.5947, 2.9592
*<CS>_M_ (Iso, Aniso)* (nm)	15.6, 15.0	23.0, 23.0	26.8, 26.2	45.0, 45.3
*<CS>_N_ (Nominal)* (nm)	14		21	
*AR*	0.85	-	0.80	-
*wt (Iso, Aniso)* %	91.8, 91.8	8.2, 8.2	88.3, 88.4	11.7, 11.6
*R_Bragg_ (Iso, Aniso)*	2.4, 1.8	2.7, 2.5	2.1, 1.6	3.5, 3.1
*R_wp_ (Iso, Aniso)*	5.2, 4.5		5.8, 4.8	

**Table 2:** Results from the Debye scattering equation (DSE) analysis of P90 and P25 using synchrotron wide-angle X-ray scattering (WAXTS) data and the bipyramids (*BIPY*) and/or prismatic *BIPY_PAR_* models for anatase nanocrystals (NCs). Averages are all mass based: equatorial width *(<L_w_>*_M_) and length (<*2L_h_*>_M_) of bipyramids, their relative dispersions (σ*_w_*/<*L_w_*>_M_ and σ*_h_*/<*2L_h_*>_M_) and correlation angle (*φ*) between growth directions. The aspect ratio (*AR*) is computed from the average width and length of the (pristine or recovered) bipyramids. {101} and {001} surface areas (as % of the total area) are calculated by summing the contribution of all NCs in the population of bipyramids, each NC weighted by its number fraction. The diameter (<*D*>_M_) and its relative dispersion (σ/<*D*>_M_) is provided for rutile; for anatase, it refers to the sphere of equivalent volume to that of bipyramids. The number-based averages values are reported in Table A2.

Parameter	P90 (BIPY)		P90 (BIPY_PAR_)		P25 (BIPY_PAR_)	
	Anatase^1^	Rutile	Anatase	Rutile	Anatase	Rutile
*<L_w_ >_M_,* (nm)	13.75	-	13.73	-	23.00	-
*<2L_h_>_M_* (nm)	13.09	-	13.18	-	20.71	-
*σ_w_/<L_w_>_M_* (×100)	37.75	-	37.75	-	41.32	-
*σ_h_/<2L_h_>_M_* (×100)	50.06	-	51.25	-	50.98	-
*φ* (°)	41.00	-	42.54	-	42.80	-
*AR = <2L_h_>_M_/<L_w_> _M_*	0.95	-	0.95	-	0.89	-
{001}, {101} surface %	21.3, 78.8	-	20.86, 79.14	-	22.73, 77.37	-
*SSA* (m^2^/g)	147.4		146.6		95.6	
*<D>_M_,* (nm) *σ/<D>_M_* (×100)	14.22, 36.07	18.13, 24.47	14.17, 36.08	18.19, 22.44	23.74, 9.68	41.85, 52.62
wt%	87.00	13.00	86.83	13.17	89.59	10.41
*B (Å^2^),* Ti, O	0.31, 0.89	0.44, 0.80	0.30, 0.84	0.44, 1.00	0.37, 0.99	
*S.o.f., Ti ^2^*	0.94	-	0.95	-	0.92	-
*GoF*	11.57		12.12		11.05	

^1^ A small fraction (amounting to ca. 20%) of the BIPY largest NCs was replaced by the prismatic shape to save computational time. NCs within the bivariate population having AR > 2.5 (0.4 wt% in P25 and 2.0 wt% in P90) were modelled as prisms.^2^ The slightly below unity values are likely due to the use of atomic form factor of neutral Ti atoms rather than the positively charged ions.

## Supplementary Materials

Supplementary figures and tables are available online at https://www.mdpi.com/2079-4991/10/4/743/s1. Figure S1: P25 and P90 Rietveld fits; Tables S1 and S2: Results from synthetic data with BIPY anatase NCs. Table S3: Surface area photocatalytic conversion of Methyl tert-butyl ether.

## Appendix A

**Table A1:** Numerical results of the BIPY_PAR_ model vs. the true BIPY values (graphically shown in Figure 3c).

	*AR* = 0.5	*AR* = 1.0	*AR* = 1.5	*AR* = 2.0
	*BIPY–BIPY_PAR_*	*BIPY–BIPY_PAR_*	*BIPY–BIPY_PAR_*	*BIPY–BIPY_PAR_*
*<L_w_>_M_* (nm)	5.83–5.80	5.59–5.59	5.19–5.22	5.11–5.12
*<2L_h_>_M_* (nm)	2.80–2.72	6.14–6.48	8.37–7.94	9.26–8.45
*σ_w_/<L_w_>_M_* (×100)	18.36–17.55	16.99–16.86	15.87–19.00	15.44–19.76
*σ_h_/<2L_h_>_M_* (×100)	23.37–24.96	23.75–33.06	23.55–33.51	22.15–33.16
*AR = <2L_h_>_M_/<L_w_>_M_*	0.48–0.47	1.10–1.16	1.61–1.52	1.81–1.65
*AR = <2L_h_/L_w_>_M_*	0.50–0.48	1.13–1.18	1.64–1.54	1.83–1.67
{001} surface area %	41.34–42.71	15.17–15.02	6.04–8.34	3.90–6.62
{101} surface area %	58.66–57.29	84.83–84.98	93.96–91.66	96.10–93.38

**Table A2:** Results from the DSE analysis of P90 and P25 using synchrotron WAXTS data and the *BIPY* and/or *BIPY_PAR_* models for anatase NCs. Averages are all number based. Entries as in Table 2.

	P90 (BIPY)		P90 (BIPY_PAR_)		P25 (BIPY_PAR_)	
	Anatase	Rutile	Anatase	Rutile	Anatase	Rutile
*<L_w_>_N_*, (nm)	8.68	-	8.76	-	9.95	-
*<2L_h_>_N_* (nm)	8.79	-	9.01	-	9.01	-
*σ_w_/<L_w_>_N_* (×100)	44.01	-	43.39	-	58.25	-
*σ_h_/<2L_h_>_N_* (×100)	54.76	-	53.33	-	62.42	-
*AR = <2L_h_>_N_/<L_w_>_N_*	1.01	-	1.03	-	0.91	-
*<D>_N_* (nm) *σ/<D>_N_* (×100)	9.06 41.67	15.21 24.61	9.14 40.87	15.68 22.54	11.72 55.61	18.90 55.50
