# Supplementary material for: Structure, Morphology, and Faceting of TiO2 Photocatalysts by the Debye Scattering Equation Method. The P25 and P90 Cases of Study
Source: Nanomaterials (Basel). 2020 Apr 13;10(4):743. doi: 10.3390/nano10040743 (PMC7221600; doi:10.3390/nano10040743)
Supplement: Supplementary file 1 [file nanomaterials-10-00743-s001.pdf]

nanomaterials

Supplementary Materials

Structure, Morphology and Faceting of TiO_2 Photocatalysts by the Debye Scattering Equation Method. The P25 and P90 Cases of Study

Federica Bertolotti[1,*], Anna Vivani[1], Daniele Moscheni[1], Fabio Ferri[1], Antonio Cervellino,[2] Norberto Masciocchi[1,*], Antonietta Guagliardi[3,*]

[1] Dipartimento di Scienza e Alta Tecnologia, To.Sca.Lab & INSTM, Università dell'Insubria, via Valleggio 11, 22100 Como (Italy)

[2] SLS, Laboratory for Synchrotron Radiation - Condensed Matter, Paul Scherrer Institut, 5232 Villigen, Switzerland

[3] Istituto di Cristallografia & To.Sca.Lab., Consiglio Nazionale delle Ricerche, via Valleggio 11, 22100 Como (Italy)

* Correspondence: antonella.guagliardi@ic.cnr.it

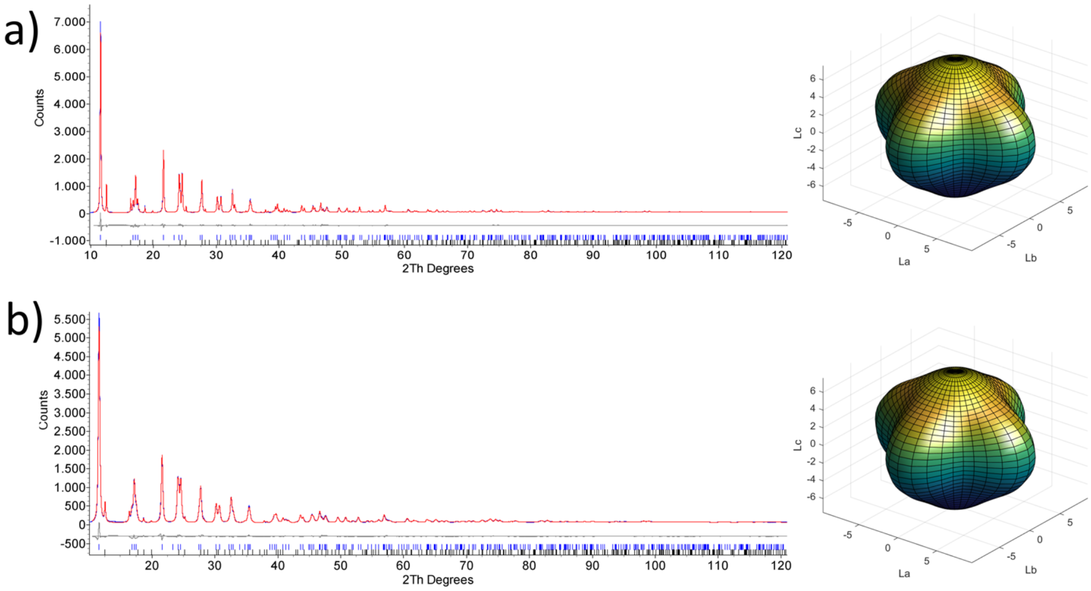

Figure S1. Left: Best fit (red curve) of the experimental WAXTS data (blue line) obtained using the conventional Rietveld approach for (a) P25 and (b) P90, according to an anisotropic morphological description of the peaks broadening for the anatase phase. Right: 3D isosurfaces describing the average crystal shapes of anatase NCs drawn on the basis of the spherical harmonics coefficients, up to the 4th order (Y_{00}; Y_{20}, Y_{40} and Y_{44}, in 4/mmm point symmetry). Insets: L_a, L_b and L_c values on the axes (in nm) are apparent crystal sizes determined by convoluting the (anisotropic) Lorentzian broadening with additional (isotropic) Gaussian broadening.

Table S1. Number (subscript *N*) and mass (subscript *M*) based averages retrieved by the $BIPY_{PAR}$ model *vs* the true BIPY parameters on which simulated patterns were built, at constant aspect ratio (*AR*=1.0) and fixed correlation angle between the two growth directions (ϕ= 0°), upon increasing the size dispersion $\sigma_w/\langle L_w\rangle_N = \sigma_h/\langle 2L_h\rangle_N$ ~ 0.1, 0.3 and 0.4 in the BIPY population (see Table A1 for $\sigma_w/\langle L_w\rangle_N$ ~ 0.2). The mass-based results are graphically shown in Figure 3d. NCs size discretization may result in small deviations from nominal values.

	$\sigma_w/\langle L_w\rangle \sim \sigma_h/\langle 2L_h\rangle \sim 0.1$	$\sigma_w/\langle L_w\rangle \sim \sigma_h/\langle 2L_h\rangle \sim 0.3$	$\sigma_w/\langle L_w\rangle \sim \sigma_h/\langle 2L_h\rangle \sim 0.4$
	BIPY – $BIPY_{PAR}$	*BIPY – $BIPY_{PAR}$*	*BIPY – $BIPY_{PAR}$*
$\langle L_w\rangle_N$ (nm)	5.20 – 5.14	5.23 – 5.25	5.28 – 5.34
$\langle 2L_h\rangle_N$ (nm)	5.87 – 6.11	5.95 – 5.86	5.80 – 5.70
$\sigma_w/\langle L_w\rangle_N$ (x100)	8.46 – 10.31	24.86 – 24.00	32.76 – 31.64
$\sigma_h/\langle 2L_h\rangle_N$ (x100)	11.58 – 27.33	34.28 – 39.59	42.59 – 45.61
$AR = \langle 2L_h\rangle_N / \langle L_w\rangle_N$	1.14 – 1.19	1.19 – 1.16	1.18 –1.14
$\langle L_w\rangle_M$ (nm)	5.30 – 5.30	6.06 – 6.07	6.77 – 6.77
$\langle 2L_h\rangle_M$ (nm)	5.89 – 6.46	6.44 – 6.56	6.69 – 6.71
$\sigma_w/\langle L_w\rangle_M$ (x100)	8.50 – 10.31	24.32 – 23.87	31.35 – 30.50
$\sigma_h/\langle 2L_h\rangle_M$ (x100)	11.57 – 27.33	33.96 – 39.47	42.22 – 45.75
$AR = \langle 2L_h\rangle_M / \langle L_w\rangle_M$	1.11 – 1.22	1.06 – 1.08	0.99 – 0.99
{001} surface area %	14.28 – 13.29	16.92 – 17.25	19.63 – 20.16
{101} surface area %	85.72 – 86.71	83.08 – 82.65	80.37 – 78.84

Table S2. Number (subscript *N*) and mass (subscript *M*) based averages retrieved by the $BIPY_{PAR}$ model *vs* the true BIPY parameters on which simulated patterns were built, at constant aspect ratio (*AR*=1.0), fixed correlation angle between the two growth directions (ϕ = 30°), constant size dispersion along the bipyramids equatorial edge ($\sigma_w/\langle L_w\rangle_N$ ~ 0.1), upon increasing the size dispersion along L_h ($\sigma_h/\langle 2L_h\rangle_N$ ~ 0.2, 0.3 and 0.4) in the BIPY population. The mass-based results are graphically shown in Figure 3f.

	$\sigma_h/\langle 2L_h\rangle$ ~ 0.2	$\sigma_h/\langle 2L_h\rangle$ ~ 0.3	$\sigma_h/\langle 2L_h\rangle$ ~ 0.4
	BIPY – $BIPY_{PAR}$	*BIPY – $BIPY_{PAR}$*	*BIPY – $BIPY_{PAR}$*
$\langle L_w\rangle_N$ (nm)	5.22 – 5.52	5.24 – 5.65	5.26 – 5.46
$\langle 2L_h\rangle_N$ (nm)	5.87 – 6.82	5.82 – 7.05	5.76 – 6.55
$\sigma_w/\langle L_w\rangle_N$ (x100)	12.84 – 8.51	17.75 – 13.63	22.62 – 21.06
$\sigma_h/\langle 2L_h\rangle_N$ (x100)	19.97– 30.79	28.35 – 31.91	36.46 – 40.00
$AR = \langle 2L_h\rangle_N / \langle L_w\rangle_M$	1.12 – 1.27	1.10 – 1.28	1.07 – 1.20
$\langle L_w\rangle_M$ (nm)	5.46 – 5.56	5.76 – 5.87	6.14 – 6.20
$\langle 2L_h\rangle_M$ (nm)	6.19 – 6.75	6.67 – 7.12	7.29 – 7.72
$\sigma_w/\langle L_w\rangle_M$ (x100)	13.0 – 8.64	17.84 – 13.78	21.85 – 20.92
$\sigma_h/\langle 2L_h\rangle_M$ (x100)	20.30 – 30.81	28.77 – 32.19	35.95 – 39.47
$AR = \langle 2L_h\rangle_M / \langle L_w\rangle_M$	1.13 – 1.21	1.16 – 1.21	1.19 – 1.25
{001} surface area %	14.27 – 13.98	14.38 –13.96	14.55 – 13.84
{101} surface area %	85.73 – 86.02	85.62 – 86.04	85.45 – 86.16

Table S3. Nitrogen gas adsorption BET (Brunauer, Emmet, Teller) surface area (S_{BET}) and S_{BET}-surface normalized photocatalytic conversion of Methyl tert-butyl ether by P90 and P25 materials from In, S. *Comparison of Photocatalytic Performance of Degussa TiO2 P25 and P90 in Gas Phase Photocatalytic Reactor*. Asian J. Chem. 2011, 23, 2629–2631.

	S_{BET} ($m^2\ g^{-1}$)	Conversion (%)	Normalized conversion (% $m^2\ g^{-1}$)
P90	100.4	72.0	0.72
P25	57.4	61.1	1.06
